# Using expert decision-making to establish indicators of urban friendliness for walking environments: a multidisciplinary assessment

**DOI:** 10.1186/s12942-016-0071-7

**Published:** 2016-11-15

**Authors:** Yen-Cheng Chiang, Han-Yu Lei

**Affiliations:** 1 Department of Landscape Architecture, National Chiayi University, Chiayi, 60004 Taiwan, ROC; 2 Department of Landscape Architecture, University of Illinois at Urbana Champaign, Champaign, IL 61820 USA; 3 Department of Horticulture, National Chiayi University, Chiayi, 60004 Taiwan, ROC

**Keywords:** Multiple-criteria decision-making, Fuzzy Delphi method (FDM), Analytic network process (ANP), Public health

## Abstract

**Background:**

Numerous studies have suggested that friendly walking environments positively affect physical activity and health. Creating friendly walking environments in urban areas is a complex and wide-ranging topic, and no study has yet established a set of assessment indicators by drawing on the expertise of various disciplines. This study uses a multiple-criteria decision-making technique to elucidate the environmental factors that affect the friendliness of the walking environment.

**Methods:**

We conducted a two-phase expert questionnaire survey. Experts from the government sector, as well as the academic disciplines of urban planning, transportation, architecture, and landscape design, were recruited to establish a set of walking environment indicators; the degrees of importance assigned to these indicators by the experts were subsequently compared. In phase 1, the fuzzy Delphi method was used by 20 experts, whose responses were used to identify four dimensions and 22 indicators. In phase 2, an analytical network process approach was performed by 16 experts to determine the weights of the dimensions and indicators.

**Results:**

The results revealed that all of the experts ranked the four dimensions in the order of safety > facilities > aesthetics > land use mix. Of the 22 indicators, land use–diversity, land use–access, sidewalk width, sidewalk continuity, and cleanliness were considered the most important.

**Conclusions:**

The results provide a reference for the management of walking environments by promoting pedestrian-oriented environments and public health.

**Electronic supplementary material:**

The online version of this article (doi:10.1186/s12942-016-0071-7) contains supplementary material, which is available to authorized users.

## Background

Walking is the most basic mode of transportation and the most common physical activity. A favorable walking environment increases physical activity, and consequently improves physical and mental health [1, 2]. The government of New York City has promoted the concept of active living by encouraging the public to incorporate walking and cycling into their daily lives, which is demonstrated by the integration of active design strategies that create friendly walking and cycling environments [3]. Previous studies have indicated that friendly walking environments improve people’s willingness to walk and pedestrians’ health [4–6]. Therefore, planning or designing an enjoyable walking environment in an urban area considerably affects the health of residents. However, although several environmental factors that influence the propensity to walk have been studied across many disciplines, there has yet to be study that uses experts from various professional disciplines to identify a set of indicators that measure the friendliness of an environment for walkers.

As a part of a built environment, walking environments typically comprise three aspects: land use, urban design, and transportation systems. Land use refers to the distributions of land (commercial or residential) with diverse uses; urban design refers to the landscape and the exterior, arrangement, and layout of buildings in a neighborhood; and transportation systems are the infrastructure or facilities that connect destinations [7, 8]. In addition, the construction of a walking environment encompasses a range of complex challenges and consists of four disciplines: urban planning, transportation, architecture, and landscape design. With respect to urban planning, a greater mixture of land uses within a region support more walking activities [6]. Kerr et al. [9] found that mixture of land uses and access to recreational spaces were significantly correlated with the walking activity levels of young people. Another study found that New York residents who lived farther from areas with pedestrian-oriented uses were less likely to engage in physical activity, and that social engagement was highly correlated with walking [4]. Therefore, increasing the accessibility of social venues enhances the probability of walking.

Regarding transportation, increasing the connectivity of the walking environment of a city encourages residents to walk to commute. Overall, street connectivity is positively correlated with the likelihood of walking as a commuting mode [10], and requires adequate sidewalks to promote walking [8]. Street networks with higher connectivity allow destinations to be more directly reached [11]. Additionally, increasing the density of street intersections with at least three legs increases the likelihood that residents walk in their neighborhoods and reduces their dependence on motor vehicles [12, 13]. In addition, some facilities, such as traffic lights and crosswalks, extend walking continuity by reducing pedestrian hazards [14]; furthermore, installing accessible ramps for those on a wheelchair or with a baby stroller expands their accessible walking distance and decreases travel time [2, 5].

With respect to architecture, pedestrians generally only notice the first floor of a building because they are typically focused on their immediate surroundings. Therefore, they tend to associate fine exterior designs of first floors, including form, texture, and building signs, with a pleasant walking experience. Moreover, the characteristics of buildings and other surroundings strongly affect the willingness of older people to walk. For example, they tend to be put off by a lack of exterior features or the insufficient maintenance of historic buildings [15].

Finally, with respect to landscape, untrimmed plants and temporary obstacles on sidewalks (such as objects placed by shops) typically divert pedestrians into traffic lanes, increasing the danger posed to them and reducing their willingness to walk [15, 16]. By contrast, high greening levels can promote walking and improve people’s mental health [17]. Additionally, Kerr et al. [18] found that when sidewalks are planted with trees, shaded by trees, or surrounded by interesting things that people can observe, or when neighborhoods include appealing natural sights, parents are more likely to allow their children to walk to school.

Multiple-criteria decision-making (MCDM) concerns the structure and resolution of decision and planning problems that involve multiple criteria, in order to support decision-makers [19]. Recently, studies have applied multiple-criteria evaluation to calculate the weights of urban area indicators that comprise the deprivation index [20], and have used local expert knowledge about social and physical factors that influence healthy food consumption to optimize potential locations [21]. However, to date, no study has established a set of indicators that assess urban walking environments according to the opinions of experts in the government or the academic disciplines of urban planning, transportation, architecture, and landscape design. Thus, this study uses a MCDM technique to elucidate the environmental factors that affect the friendliness of walking environments, discusses the importance of each factor, and compares the importance levels that are assigned to the indicators by experts from various disciplines.

## Methods

**Fig. 1:**
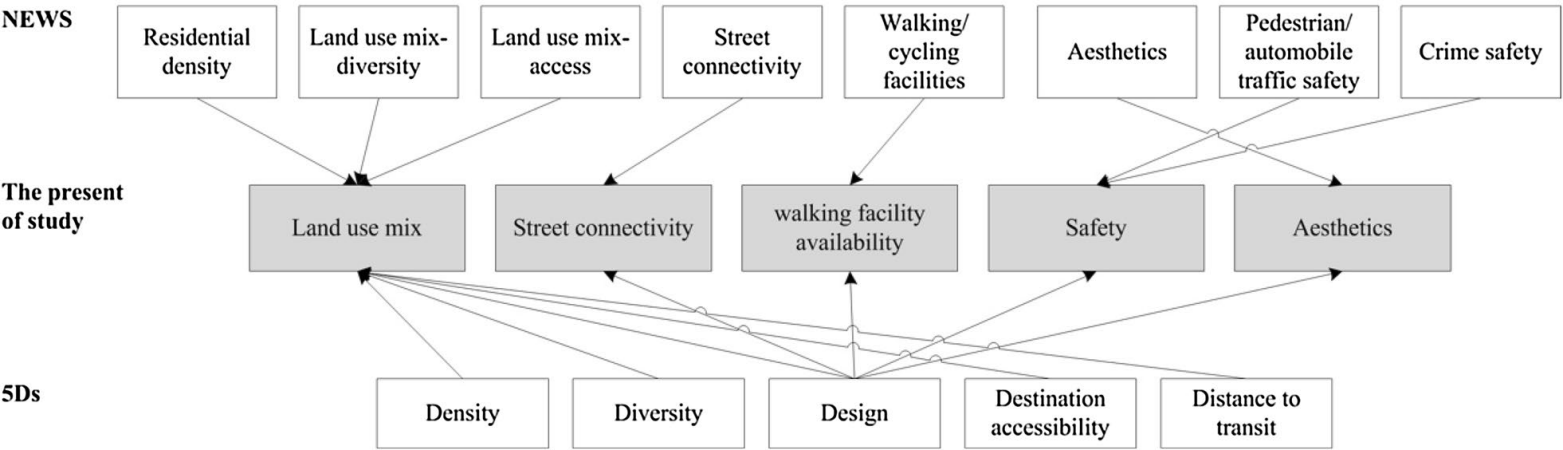
Five dimensions of this study

 To establish the dimensions of a walking environment and the indicators of its friendliness, the Neighborhood Environment Walkability Scale (NEWS) developed by Saelens et al. [2] and the 5Ds developed by Cervero et al. [22] were used. Verified by numerous studies, the NEWS is a highly validated and reliable scale of self-reported walking [23–25] that considers eight environmental characteristics: residential density, land use mix–diversity, land use mix–access, street connectivity, walking and cycling facilities, aesthetics, pedestrian and automobile traffic safety, and crime safety. The 5Ds comprise density, diversity, design, destination accessibility, and distance to transit. Density refers to the density of the population or buildings, and diversity refers to the degree of mixing land uses. Design refers to (1) walking facilities, including the density of roadside trees and traffic lights; (2) street connectivity; and (3) the safety of the walking environment. Destination accessibility refers to the number of libraries, schools, medical institutions, and stations that can be reached by walking, and finally, distance to transit is the distance between a household and public transportation stations. According to the concepts of the NEWS and 5Ds, five dimensions (land use mix, street connectivity, availability of walking facilities, safety, and aesthetics) were developed and used as dimensions in this study (Fig. 1).

To solve the complicated concerns in a walking environment, a two-phase expert questionnaire survey was administered. First, experts’ evaluations were compiled using the fuzzy Delphi method (FDM), a type of MCDM, for reducing the number of questionnaire rounds normally necessary to obtain interdisciplinary consensus and sift through the dimensions and indicators [26]. Subsequently, the weight of each dimension and indicator was assigned using an analytic network process (ANP). The procedures of these two phases are described in the following sections.

- *Phase 1* Selection of friendliness indicators of urban walking environments.

This phase consisted of the selection of indicators through an expert questionnaire survey using the FDM. After describing the friendliness indicators of walking environments, the experts assigned levels of importance to them. As a method that was developed to overcome the weaknesses of the conventional Delphi method by applying fuzzy theory [27], the FDM can be used to analyze the fuzzy concepts of human minds and resolve uncertainty in subjective judgments. Thus, the FDM is more effective than the conventional Delphi method at selecting an optimal set of indicators, and can reduce the number of surveys that must be performed to elicit relatively complete expert opinions [28]. Phase 1 comprised the following four procedures.

1. Establishing a framework and indicator descriptions.

The aforementioned five dimensions were associated with a total of 30 indicators. Specifically, land use mix refers to land use mix–diversity, land use mix–access, and population density; street connectivity comprises intersection density, dead-end street density, and alternative routes; availability of walking facilities refers to sidewalk material, wayfinding aids, pedestrian squares, barrier-free designs, sidewalk maintenance, sidewalk width, protective equipment against the weather, and amenities; safety consists of sidewalk continuity, sidewalk obstructions, sidewalk visibility, parking spaces for motor vehicles and bicycles, street lighting, buffers between roads and sidewalks, pedestrian crossing aids, traffic control facilities, bicycle lanes, and fear of crime; finally, aesthetics refers to green ratio, building attractiveness, historical landscape, cleanliness, presence of trees, and natural sights. Further details about the dimension and indicators used in this study are compiled in the Supplementary Materials (Additional file 1: Tables S1 and S2). An assigned researcher directly interpreted each dimension and indicator with the experts to avoid ambiguity and consequent errors.

2. Formation of the expert panel.

Before the questionnaire survey was performed, experts were identified on the basis of their areas of specialization, familiarity with this research topic, and authority in the field. Each recruited expert was at least one of the following: (1) an administrator who plans or manages affairs related to this research topic, (2) a researcher that explores similar topics, (3) an expert in a similar research area or topic, or (4) a specialist that has published articles related to walkability. Factors that influence walking environments are associated with the diverse disciplines of landscape design, transportation, architecture, and urban planning; thus, to elicit a wide range of professional opinions, 20 experts from the government and academic sectors of Taiwan were recruited as survey respondents. The participants comprised 17 university professors or researchers with doctoral degrees (of whom five specialized in landscape design, four in transportation, four in architecture, and four in urban planning) and three experts with Master’s degrees from Taipei City’s Department of Urban Development. According to Dalkey [29], group error can be reduced and group reliability can be maximized by using a panel with at least ten members.

3. Distribution of questionnaires.

An expert questionnaire survey using the FDM was distributed between April and May 2015, and a total of 20 were returned. A statistical analysis revealed that the experts’ opinions concerning 13 indicators did not converge; therefore, the survey was redistributed between May and June of the same year, and 20 questionnaires were again returned.

4. FDM.

The steps of FDM were as follows [28, 30].

**Fig. 2:**
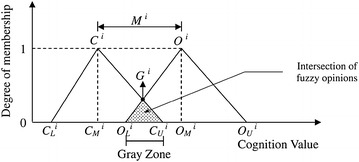
Two triangular fuzzy numbers *Source*: Chan et al. [30]
- If the fuzzy trigonometric values do not overlap ($C_{U}^{i} \le O_{L}^{i}$), then the interval values provided by the individual experts have a consensus section, and the opinion of each expert tends to fall in the consensus interval. Therefore, the “consensus degree of importance” ($G^{i}$) of the value $i$ equals the arithmetic mean of $C_{M}^{i}$ and $O_{M}^{i}$, expressed as $G^{i} = \left( {C_{M}^{i} + O_{M}^{i} } \right)/2$.
- If two of the fuzzy trigonometric values overlap ($C_{U}^{i} > O_{L}^{i}$), and the gray zone of the fuzzy relation $Z^{i} = C_{U}^{i} - O_{L}^{i}$ is smaller than the interval range of the “optimistically cognitive geometric mean” and the “conservatively cognitive geometric mean” of the experts’ evaluated project $Mi = O_{M}^{i} - C_{M}^{i}$, this indicates that although there is no consensus on the interval value section from each expert, the two experts who gave extreme views (i.e., the most conservative of the optimistic cognitive value, and the most optimistic of the conservative cognitive value) did not differ from the opinions of the other experts, which caused the variation in opinion. Hence, the “cognitive importance degree of value” ($G^{i}$) is equal to the fuzzy collection minimum when calculated from the fuzzy relation of the two trigonometric values; thus, the quantified value of the fuzzy collection with the maximum degree of membership is obtained.
- If two of the fuzzy trigonometric values overlap $(C_{U}^{i} > O_{L}^{i} )$, and the gray zone of the fuzzy relation $Z^{i} = C_{U}^{i} - O_{L}^{i}$ is larger than the interval range of the “optimistically cognitive geometric mean” and the “conservatively cognitive geometric mean” of the experts’ evaluated project $Mi = O_{M}^{i} - C_{M}^{i}$, then there is no consensus on interval value section from each expert; thus, the two experts who gave the most extreme views differ from the other experts, which leads to a divergence of opinion. The evaluated values are then collected for the opinions that do not converge and provided to the experts as the reference, Steps 1–4 are repeated, and a new questionnaire is produced until the entire evaluated project receives convergence to acquire the consensus importance value $G^{i}$.
- *Phase 2* Establishment of the weights of friendliness indicators of urban walking environments.

- **Step 1** Collect an interval value of each assessment indicator from each expert. Notably, the minimum interval value represents an expert’s “most conservative cognition value,” whereas the maximum interval value represents an expert’s “most optimistic cognition value” of the assessment indicator.
- **Step 2** Perform a statistical analysis on each expert’s most conservative cognitive value and most optimistic cognitive value for each interval value $i$. Extreme values that were more than two standard deviations from the mean were excluded. Subsequently, the minimum value $C_{L}^{i}$, geometric mean $C_{M}^{i}$, and maximum value $C_{U}^{i}$ of the remaining most conservative cognitive values, as well as the minimum value $O_{L}^{i}$, geometric mean $O_{M}^{i}$, and maximum value $O_{U}^{i}$ of the remaining most optimistic cognitive values were calculated.
- **Step 3** Establish the trigonometric fuzzy value $C^{i} = \left( {C_{L}^{i} , C_{M}^{i} ,C_{U}^{i} , } \right)$ from the most conservative cognitive value and the trigonometric fuzzy value $O^{i} = \left( {O_{L}^{i} , O_{M}^{i} ,O_{U}^{i} , } \right)$ from the most optimistic cognitive value for each interval value $i$ that was calculated in Step 2.
- **Step 4** Examine whether the experts have reached a consensus using the following measures (Fig. 2):

According to the expert questionnaire survey results of the phase 1 FDM, 22 friendliness indicators of walking environments were selected and the ANP was used to establish the weights of the indicators. In contrast with the analytic hierarchy process, the ANP includes feedback in the decision-making process that captures interdependence among the variables and generates a decision-making process that is relatively close to reality [31, 32]. Therefore, the weight of each indicator was calculated using Super Decisions 2.2.6, a decision-making software developed by the Creative Decisions Foundation [33].

1. Expert panel.

To prevent the experts from misunderstanding the survey content, and to ensure consistent results from the surveys that used the FDM and ANP techniques, the questionnaire surveys of both phases were distributed to the same 20 respondents. The ANP questionnaires were distributed and returned in July 2015. A total of 16 valid questionnaires were returned, yielding a response rate of 80%.

2. Pairwise comparison matrices and priority vectors.

A pairwise comparison of the dimensions and indicators obtained in phase 1 was conducted to determine the priority vector. Similar to an analytic hierarchy process [20], the dimensions were compared using the importance levels that were assigned to the selected indicators, and the indicators were compared by their influence levels among all the indicators. Accordingly, the dimensions or indicators that were most critical to the creation of friendly urban walking environments were identified. In addition, reciprocal values were used to produce pairwise comparisons in which the elements were reversed, as seen in Formula (1). Pairwise comparisons using the ANP approach were made to generate initial priority vectors of the pairwise comparison matrices, and to evaluate the magnitude of the interdependence between each element or matrix.

1 $$a_{ij} = \frac{1}{{a_{ij} }}$$

3. Supermatrix formation.

To generate global priorities in a system with interdependent influences, the obtained local priority vectors and matrices from a step pairwise comparison were entered into a matrix to form a “supermatrix” according to the standard supermatrix form provided by Saaty and Vargas [32]. After the supermatrix was formed, a weighted supermatrix was obtained by transforming the sum of each column into unity, in a manner similar to using a Markov chain to ensure a column–stochastic matrix [31]. Subsequently, to achieve a convergence for the weights, the weighted supermatrix was raised to its limiting powers [as seen in Formula (2)] to obtain the limit supermatrix [34, 35] and reveal the long-term stable weighted values and global priority weights [36]. Saaty [34] also provided further details about the mathematical procedure of the ANP approach.

2 $$\mathop {\lim }\limits_{k \to \infty } W^{2k + 1}$$

## Results

### Selection of indicators using the FDM

After the expert questionnaire survey in phase 1 was returned, Microsoft Excel 2010 was used to determine the overlapping of the trigonometric fuzzy values of the following 13 indicators: land use mix–diversity, population density, intersection density, dead-end street density, alternative routes, sidewalk material, wayfinding aids, pedestrian squares, protective equipment against the weather, traffic control facilities, bicycle lanes, historical landscape, and natural sights. The results suggested that the experts did not reach a consensus, and that the extreme values assigned by some experts differed greatly, reflecting a divergence of opinions. Therefore, the phase 1 survey was administered again, and involved the 13 divergent indicators in an attempt to obtain a consensus on the crucial values from the experts.

After the survey was redistributed, the trigonometric fuzzy values of the indicators were obtained and a gray zone test was conducted to determine whether the experts’ opinions converged; notably, substantial consensus among importance values would indicate that the experts assigned high importance levels to the same indicators. A threshold value was set before the selection of the indicators was finalized, on the basis of Klir and Folger’s [37] suggestion that an ideal threshold value is typically between six and seven. In the present study, the threshold value was set at 6.12, and the following four indicators were removed: population density (from the land use mix dimension), and intersection density, dead-end street density, and alternative routes (from the street connectivity dimension). Additionally, because Saaty [34] suggested that no more than seven indicators be offered under each dimension, we removed another four that had with lower consensus importance values: pedestrian squares (from the availability of walking facilities dimension), and parking spaces for motor vehicles and bicycles, traffic control facilities, and bicycle lanes (from the safety dimension). The remaining 22 indicators were analyzed by an ANP for weighting (Table 1).

**Table 1:** Indicator selection

Goal	Dimension (D)	Indicator (I)	Consensus importance value	Removed/retained
Friendly urban walking environments	D1 Land use mix	I1-1 Land use mix–diversity	6.27	Retained
		I1-2 Land use mix–access	6.78	Retained
		I1-3 Population density	5.61	Removed
	D2 Street connectivity	I2-1 Intersection density	5.54	Removed
		I2-2 Dead-end street density	5.50	Removed
		I2-3 Alternative routes	5.97	Removed
	D3 Availability of walking facilities	I3-1 Sidewalk material	7.40	Retained
		I3-2 Wayfinding aids	6.87	Retained
		I3-3 Pedestrian squares	6.38	Removed
		I3-4 Barrier-free design	7.97	Retained
		I3-5 Sidewalk maintenance	7.18	Retained
		I3-6 Sidewalk width	8.39	Retained
		I3-7 Protective equipment against the weather	6.92	Retained
		I3-8 Amenities	6.82	Retained
	D4 Safety	I4-1 Sidewalk continuity	8.00	Retained
		I4-2 Sidewalk obstructions	7.51	Retained
		I4-3 Sidewalk visibility	7.70	Retained
		I4-4 Parking space for motor vehicles and bicycles	6.12	Removed
		I4-5 Street lighting	7.19	Retained
		I4-6 Buffer between road and sidewalk	7.38	Retained
		I4-7 Pedestrian crossing aids	7.05	Retained
		I4-8 Traffic control facilities	6.45	Removed
		I4-9 Bicycle lanes	6.68	Removed
		I4-10 Fear of crime	6.72	Retained
	D5 Aesthetics	I5-1 Green ratio	6.92	Retained
		I5-2 Building attractiveness	6.24	Retained
		I5-3 Historical landscape	7.34	Retained
		I5-4 Cleanliness	7.11	Retained
		I5-5 Presence of trees	6.71	Retained
		I5-6 Natural sights	7.00	Retained

### Determining indicator weighting using an ANP approach

After the responses from the 16 experts were compiled, Super Decisions 2.2.6 [33] was used to calculate the unweighted supermatrices of the remaining indicators. Specifically, we multiplied the matrix from each dimension by the unweighted supermatrices of the indicators to yield the weighted supermatrices, which then underwent limit multiplication. The weight values in the matrices converged to a fixed value, yielding limited supermatrices that were subsequently used to calculate the overall relative weight of each indicator. The results revealed that, according to the weight values, the four dimensions ranked as follows: safety > availability of walking facilities > aesthetics > land use mix (Tables 2, 3).

**Table 2:** Dimension and indicator weighting

Dimension	Weight	Rank	Indicator	Weight	Rank
Land use mix	0.07	4	Land use mix–diversity	0.50	1
			Land use mix–access	0.50	1
Availability of walking facilities	0.31	2	Sidewalk material	0.13	4
			Wayfinding aids	0.10	6
			Barrier-free design	0.20	2
			Sidewalk maintenance	0.17	3
			Sidewalk width	0.22	1
			Protective equipment against weather	0.10	5
			Amenities	0.08	7
Safety	0.50	1	Sidewalk continuity	0.21	1
			Sidewalk obstructions	0.14	3
			Sidewalk visibility	0.16	2
			Street lighting	0.14	4
			Buffer between road and Sidewalk	0.11	7
			Pedestrian crossing aids	0.11	6
			Fear of crime	0.12	5
Aesthetics	0.12	3	Green ratio	0.17	4
			Building attractiveness	0.13	5
			Historical landscape	0.12	6
			Cleanliness	0.20	1
			Presence of trees	0.19	2
			Natural sights	0.18	3

**Table 3:** Weight and ranking of each dimension based on expert opinions

Dimension	Weight (Rank)				
	Urban planning	Transportation	Architecture	Landscape	Government
Land use mix	0.07 (4)	0.07 (4)	0.10 (4)	0.07 (4)	0.06 (4)
Availability of walking facilities	0.41 (2)	0.21 (2)	0.23 (2)	0.24 (2)	0.42 (2)
Safety	0.42 (1)	0.52 (1)	0.50 (1)	0.59 (1)	0.43 (1)
Aesthetics	0.11 (3)	0.20 (3)	0.18 (3)	0.10 (3)	0.09 (3)

Notably, both indicators in the land use mix dimension (land use mix–diversity and land use mix–access) attained an equal weight value. In the walking facilities dimension, the sidewalk width, barrier-free design, and sidewalk maintenance were the three most crucial indicators. The key indicators in the safety dimension were sidewalk continuity, sidewalk visibility, and sidewalk obstructions. Finally, cleanliness, the presence of trees, and natural sights were the prominent indicators in the aesthetics dimension, whereas historical landscape was the least important indicator. A summary of these results is presented in Table 2.

Each indicator was weighted based on the experts’ responses. Regarding the land use mix dimension, the urban planning experts considered land use mix–diversity to be the more essential indicator, whereas the transportation, architecture, landscape design, and government experts regarded land use mix–access to be the more essential indicator (Table 4). Concerning the availability of walking facilities dimension, the urban planning, transportation, and landscape design experts considered a barrier-free design, sidewalk maintenance, and sidewalk width to be more crucial than the other indicators, whereas the architecture experts assessed sidewalk maintenance, sidewalk width, and protective equipment against the weather to be more crucial than a barrier-free design; by contrast, the government experts indicated that a barrier-free design, sidewalk width, and sidewalk material are more critical than the other indicators.

**Table 4:** Weight and ranking of each indicator based on expert opinions

Indicator	Weight (rank)				
	Urban planning	Transportation	Architecture	Landscape	Government
Land use mix–diversity	0.57 (1)	0.40 (2)	0.40 (2)	0.50 (1)	0.47 (2)
Land use mix–access	0.43 (2)	0.60 (1)	0.60 (1)	0.50 (1)	0.53 (1)
Sidewalk material	0.10 (5)	0.12 (5)	0.10 (7)	0.15 (4)	0.19 (3)
Wayfinding aids	0.12 (4)	0.13 (4)	0.13 (5)	0.07 (6)	0.08 (5)
Barrier-free design	0.21 (2)	0.21 (1)	0.12 (6)	0.21 (1)	0.23 (1)
Sidewalk maintenance	0.15 (3)	0.14 (3)	0.16 (2)	0.21 (1)	0.14 (4)
Sidewalk width	0.25 (1)	0.20 (2)	0.16 (2)	0.21 (1)	0.23 (1)
Protective equipment against weather	0.10 (5)	0.10 (6)	0.19 (1)	0.08 (5)	0.07 (6)
Amenities	0.08 (6)	0.09 (7)	0.15 (4)	0.07 (6)	0.06 (7)
Sidewalk continuity	0.21 (1)	0.18 (2)	0.10 (6)	0.26 (1)	0.23 (1)
Sidewalk obstructions	0.18 (2)	0.16 (3)	0.09 (7)	0.16 (2)	0.13 (4)
Sidewalk visibility	0.11 (5)	0.19 (1)	0.14 (4)	0.14 (3)	0.23 (1)
Street lighting	0.09 (7)	0.14 (5)	0.17 (2)	0.13 (4)	0.15 (3)
Buffer between road and sidewalk	0.12 (4)	0.09 (6)	0.14 (4)	0.11 (6)	0.10 (5)
Pedestrian crossing aids	0.10 (6)	0.09 (6)	0.15 (3)	0.12 (5)	0.08 (6)
Fear of crime	0.17 (3)	0.15 (4)	0.21 (1)	0.08 (7)	0.08 (6)
Green ratio	0.19 (2)	0.17 (3)	0.12 (6)	0.15 (4)	0.23 (1)
Building attractiveness	0.12 (5)	0.12 (6)	0.15 (4)	0.10 (6)	0.13 (5)
Historical landscape	0.17 (3)	0.15 (5)	0.15 (4)	0.11 (5)	0.10 (6)
Cleanliness	0.17 (3)	0.17 (3)	0.21 (1)	0.23 (2)	0.17 (3)
Presence of trees	0.11 (6)	0.19 (1)	0.16 (3)	0.24 (1)	0.22 (2)
Natural sights	0.23 (1)	0.19 (1)	0.20 (2)	0.16 (3)	0.15 (4)

Examining the safety dimension indicators revealed that the landscape design and transportation experts regarded sidewalk continuity, sidewalk obstructions, and sidewalk visibility to be more crucial than the others, whereas the urban planning experts assessed sidewalk continuity, sidewalk obstructions, and fear of crime to be more critical than the other indicators. The architecture experts also had a different perspective, reporting that fear of crime, street lighting, and pedestrian crossing aids are more important than the other indicators; additionally, the government experts considered sidewalk continuity, sidewalk visibility, and street lighting to be more important than the other indicators. Finally, regarding the aesthetic dimension, the transportation, architecture, and landscape design experts perceived cleanliness, presence of trees, and natural sights to be more important than the other indicators, whereas the urban planning experts emphasized the importance of natural sights and the green ratio; moreover, the government experts viewed the green ratio, presence of trees, and cleanliness to be more essential than the other indicators. An outline of these results is summarized in Table 4.

## Discussion

In this study, a two-phase expert questionnaire survey was administered to identify environmental factors that influence the friendliness of urban walking environments. From the experts’ responses, the weight of each dimension and indicator was calculated, and the importance and ranking of the indicators were determined. Furthermore, the study results prompted the following discussion.

First, when the original five dimensions and 30 indicators were reduced to four dimensions and 22 indicators using the FDM in phase 1, the entire street connectivity dimension was removed because its three indicators had low consensus importance values. This perhaps reflects how street connectivity focuses on streets, rather than sidewalks, although one previous study suggested that fewer dead-end streets in a neighborhood corresponds with a greater number of people traveling on foot [38]. Furthermore, a high street density may correlate with more traffic accidents, which could be responsible for the lower consensus importance values that were assigned to the indicators of the street connectivity dimension.

Second, the experts’ responses individually and collectively revealed that safety was the most critical dimension, followed by the availability of walking facilities, aesthetics, and land use mix. Therefore, safety is a critical factor in determining whether urban walking environments are favorable. A walking environment should provide basic protection to pedestrians, because a walking environment that is not designed safely may be associated with more street accidents and reduce pedestrians’ willingness to walk. The weighting of each indicator revealed that diversity and access were equally important land use mix indicators, and that sidewalk width and maintenance and a barrier-free design were the most crucial indicators concerning the availability of walking facilities. Because the accessibility of a walking environment determines whether pedestrians can reach their destinations, limited sidewalk width, the lack of a barrier-free design (e.g., the inclusion of roadside ramps), and potholes that form following insufficient road maintenance, can prevent neighborhood residents from using nearby sidewalks. Well-designed curb ramps are not only beneficial for older citizens, but also for people with physical disabilities [15]. Other barrier-free designs, such as sloped ends, curbs constructed with adequate materials, tactile paving that is designed to benefit the visually impaired, and audio traffic lights, can also help the people with physical disabilities safely navigate through their environment.

Furthermore, sidewalk continuity, visibility, and obstructions were considered to be the most critical indicators of safety. Roadside discontinuity prevents pedestrians from reaching destinations and can even divert them onto traffic lanes, which jeopardizes their safety. Vendors, building signs, or bicycles that are parked on the roadside can also affect pedestrians’ ability to see the sidewalk and thus reduce mobility. Finally, cleanliness, the presence of trees, and natural sights were regarded as the most important aesthetic indicators. Sidewalks that are clean or have natural sights, such as trees or rivers, not only provide a sense of beauty, but also provide a comfortable and pleasant walking experience for pedestrians.

Third, the weighting of the land use mix indicators assigned by urban planning experts differed moderately from that provided by the experts of other fields. Specifically, the urban planning experts regarded diversity as the key indicator, whereas all the other experts considered access to be the most critical indicator. Because urban planning research generally focuses on diverse land development, we inferred that experts in this discipline tend to regard substantial diversity in land use as more essential than highly accessible land, as well as more conducive to increasing pedestrians’ willingness to walk. By contrast, the other experts argued that a shorter distance to destinations (e.g., commercial districts, schools, banks, and shops) increased mobile efficiency, and thus accessibility is more imperative.

Fourth, all of the experts had similar opinions concerning the indicators associated with the availability of walking facilities, and considered sidewalk width and a barrier-free design as the most crucial indicators. Sufficient sidewalk width is calculated by the total sidewalk width minus the width of obstructions. Because an adequate walking environment must satisfy the requirements of various users, sidewalk designers must consider pedestrian, lingering, and frontage zones. All of the experts also agreed that designers must incorporate a universal design into sidewalks to increase the ease of use by pedestrians of various groups and ages, and with various needs.

Fifth, most of the experts considered sidewalk continuity and visibility to be more critical than the other safety indicators. However, the architecture experts assigned the highest importance to fear of crime, which refers to the degree of concern that a crime may be committed against a pedestrian. One study demonstrated that public buildings can be designed to reduce crime rates [39]; additionally, continuity, cleanliness, and durability of public spaces can also help to prevent crime, which perhaps explains why the architecture experts placed more emphasis on the fear of crime indicator.

Sixth, cleanliness was regarded as the most critical aesthetic indicator, followed by the presence of trees, the green ratio, and natural sights. Aesthetics is necessary, because developing an adequate walking environment without improving cleanliness can negatively impact the walking environment. In particular, all of the experts considered the presence of trees and overall green space as necessary for an adequate walking environment.

Finally, most of the experts also assigned the same weighting to the indicators with only a few exceptions. Additionally, experts tended to assign greater importance to the indicators that were closely associated with their area of expertise.

## Recommendations

### Recommendations for future research

The results of this study yielded two primary recommendations for future studies. First, the findings herein were obtained by identifying adequate indicators and obtaining their weighted value. We therefore suggest that the same indicators should be used to develop questionnaires for the public. Respondents’ perceptions of walking environments can then be analyzed to facilitate the practical evaluation of urban walking environments, and to explore new directions associated with this topic. Second, in addition to excluding inadequate indicators, this study generated an indicator framework according to the results of previous studies; thus, we were unable to include all possible dimensions and indicators. We suggest that future studies should involve in-depth expert interviews to generate a more comprehensive indicator framework.

### Practical implications

Establishing a set of indicators for urban walking environments can help policymakers efficiently create and manage friendly urban walking environments. Our findings endorse the following practical suggestions:

1. Safety should be the top priority when improving walking environments. As this study noted, sidewalk continuity, visibility, and obstruction are critical safety indicators; in addition, we argue that reducing traffic speed limits can increase pedestrians’ sense of safety. The most common problem in an urban area is the poor sidewalk continuity between old and new neighborhoods, and multiple phases may be required to solve this problem. For example, sidewalks may initially be partially constructed or constructed on only one side of the road, and later followed by a connection of traffic lights or crosswalks for continuity. In addition, reducing sidewalk obstructions can maintain smoothness by ensuring an adequate sidewalk width.
2. Sidewalk width, a barrier-free design, and sidewalk maintenance were considered to be the most important indicators of available walking facilities. We suggest the following goals to improve these factors: (1) Increase effective sidewalk width, which must be achieved to successfully reduce the problems associated with sidewalk obstructions. However, if an otherwise adequate sidewalk width is overcrowded by the obstructions of surrounding merchants, the effective sidewalk width will still be rendered inadequate. (2) Install curb ramps, tactile paving, and audio traffic lights to assist people with physical disabilities.
3. Aesthetically, cleanliness and the presence of trees are crucial indicators for walking environments. In addition to regular cleaning by janitors at the governmental level, local youth employment programs could be implemented during holidays or summer breaks to maintain the cleanliness. These programs would emphasize that keeping communities trash-free is not just a cleanliness concern, but is also a feature of public health and quality of life that affects all living things. Furthermore, these programs could encourage community participation in public affairs, and alleviate pressure on the government. Although sidewalk trees increase the aesthetics of the streets, falling leafs and fruit may cause cleanliness problems or pedestrian hazards; therefore, the surrounding environment must be carefully reviewed for its feasibility of growing sidewalk trees. Finally, instead of employing enforcement or installing adequate lighting (i.e., “eyes on the street”) to prevent graffiti, a budget could be allocated for the development of street arts and the graffiti subculture. Consequently, graffiti arts may also transform the urban landscape and help shed its negative image.
4. Diversity and access were regarded as equally critical land use indicators. As part of comprehensive walking environment planning, policymakers should therefore consider the classification of land uses, and carefully examine the distance between pieces of land with various uses, including land associated with public transportation stations, open public spaces, commercial districts, markets, and schools.
5. Finally, improving walking environments requires a substantial amount of investment from competent authorities. However, for cost efficiency, we can first focus on improving existing facilities, such as lighting, ramps, and crossings, and enhancing sidewalk cleanliness and pavement maintenance. From a massive budget perspective, urban planning is the first challenge in the walkability of a city, and a determinant factor in urban planning policy is zoning. This concept determines not only the structure of the area, but also access to public transit, business areas, and recreational facilities. Subsequently, detailed infrastructure could be introduced to formulate a comprehensive and friendly walking environment.

## Conclusion

In this study, we conducted a two-phase questionnaire survey with experts from various disciplines. The findings suggested that all experts ranked safety as the most critical dimension, followed by availability of walking facilities. Of the indicators, land use–diversity, land use–access, sidewalk width, sidewalk continuity, and cleanliness were considered the most important. The results provide a reference for the management of walking environments by promoting pedestrian-oriented environments and public health.

## Additional file

**Additional file 1.** Description of urban walkability dimensions and indicators.

## Abbreviations

ANP: analytic network process
FDM: fuzzy Delphi method
MCDM: multiple-criteria decision-making
NEWS: neighborhood environment walkability scale
