# Supplementary material for: Using expert decision-making to establish indicators of urban friendliness for walking environments: a multidisciplinary assessment
Source: Int J Health Geogr. 2016 Nov 15;15:40. doi: 10.1186/s12942-016-0071-7 (PMC5111263; doi:10.1186/s12942-016-0071-7)
Supplement: Supplementary file 1 — Additional file 1. Description of urban walkability dimensions and indicators. [file 12942_2016_71_MOESM1_ESM.docx]

**Supplementary Materials**

**Table S1** Initially proposed dimensions

| Dimension | Description |
| --- | --- |
| Land use mix | Distribution of land by use, including residential, commercial, and recreational uses, and schools. |
| Street connectivity | Directness of routes between destinations - generally determined by the density of street intersections with at least three legs and the number of dead-ends. |
| Availability of walking facilities | Sidewalk materials, maintenance status, sidewalk width, and sidewalk designs that facilitate use by the physically challenged. |
| Safety | Facilities to protect walking pedestrians. Such facilities include wide and continuous sidewalks, separators of flowing traffic, and traffic lights that help pedestrians to cross streets. |
| Aesthetics | Overall aesthetics of built environments and natural sights. Built environments include exteriors of buildings and public facilities. Natural sights include rivers, lakes, and street green spaces that provide a pleasant walking experience. |

**Table S2** Initially proposed indicators

| Dimension | Indicator | Description | References |
| --- | --- | --- | --- |
| Land use mix | Land use mix–diversity | Numbers of transportation destinations (including parking lots and public transit stations), open public spaces (including parks, beaches, and playgrounds), recreational areas (including outdoor athletic fields, swimming pools, stadiums, and gyms), government or public service buildings \ (including art museums, police stations, medical service institutions, elementary schools, post offices, and libraries), commercial destinations (including convenience stores, supermarkets, marketplaces, clothes shops, and restaurants), and amenities (including public benches and toilets). | [4, 9, 11] |
|  | Land use mix–access | Distance between transportation destinations (including parking lots and public transit stations), open public spaces (including parks, beaches, and playgrounds), recreational areas (including outdoor athletic fields, swimming pools, stadiums, and gyms), government or public service buildings (including art museums, police stations, medical service institutions, elementary schools, post offices, and libraries), commercial destinations (including convenience stores, supermarkets, marketplaces, clothes shops, and restaurants), and amenities (including public benches and toilets). | [4, 10, 40] |
|  | Population density | Population per hectare. In this paper, this term specifically refers to net population density. | [13, 41] |
| Street connectivity | Intersection density | Number of street intersections with at least 3 legs. | [42] |
|  | Dead-end street density | Number of dead-ends. | [2, 13, 22, 42] |
|  | Alternative routes | Number of routes to a destination. | [2] |
| Availability of walking facilities | Sidewalk material | Materials used on sidewalk surfaces to meet pedestrians’ requirements. | [8, 25] |
|  | Wayfinding aids | Road signs with street names that can be clearly seen from sidewalks. | [8, 25] |
|  | Pedestrian squares | Number of pedestrian squares. | [15, 16] |
|  | Barrier-free design | Provision of barrier-free sidewalk facilities, such as wheelchair ramps, tactile paving, and audio traffic lights. | [2, 5] |
|  | Sidewalk maintenance | Maintenance status of sidewalks with bumps, cracks, and holes. | [43-45] |
|  | Sidewalk width | Effective sidewalk width. | [16] |
|  | Protective equipment against weather | Equipment that provides pedestrians shelter from weather conditions and a comfortable walking experience. | [43] |
|  | Amenities | Public facilities that include trash cans, public seats, and public phone booths. | [16] |
| Safety | Sidewalk continuity | Consistent continuity of sidewalks. | [6, 10, 11, 42] |
|  | Sidewalk obstructions | Permanent obstructions (such as obtruding parts of buildings, light posts, road signs, poles, trash cans, and variations in level of sidewalk) and temporary obstructions (such as illegally parked motor vehicles and illegally located vendors). | [15] |
|  | Sidewalk visibility | Degree of visual obstruction (for pedestrians) caused by nearby buildings, roadside vehicles, and vegetation. | [46] |
|  | Parking spaces for motor vehicles and bicycles | Sidewalk parking spaces for motor vehicles and bicycles, such as roadside parking grids, ground-level parking lots, and bicycle racks. | [10, 11] |
|  | Street lighting | Lighting condition of sidewalks. | [5, 46] |
|  | Buffers between roads and sidewalks | Buffer spaces between roads and sidewalks. | [7, 47] |
|  | Pedestrian crossing aids | Pedestrian crossing signs, traffic islands, curb extensions, pedestrian overpasses and underpasses, and crosswalks. | [43, 44] |
|  | Traffic control facilities | Traffic lights, speed bumps, and chicanes in traffic lanes. | [43, 44] |
|  | Bicycle lanes | Bicycle lanes clearly distinguished from traffic lanes and sidewalks. | [2, 43] |
|  | Fear of crime | Social order in the neighborhood. | [48] |
| Aesthetics | Green ratio | Ratio of green plants seen at eye level by pedestrians. | [7] |
|  | Building attractiveness | Roadside buildings with distinctive features, street art, and attractiveness of public facilities. | [7] |
|  | Historical landscape | Number of historical landscapes. | [7, 11] |
|  | Cleanliness | Degree of cleanliness, as determined by the absence of trash, pet waste, fallen leaves, and graffiti on sidewalks and building exteriors. | [7, 48, 49] |
|  | Presence of trees | Attractive street trees on sidewalks. | [7, 48, 49] |
|  | Natural sights | Visually attractive streams, rivers, lakes, and mountains that pedestrians can see from a sidewalk. | [49, 50] |
